# Perchlorate Fusion–Hydrothermal Synthesis of Nano‐Crystalline IrO_2_: Leveraging Stability and Oxygen Evolution Activity

**DOI:** 10.1002/smll.202412237

**Published:** 2025-03-30

**Authors:** Genevieve C. Moss, Tobias Binninger, Ziba S. H. S. Rajan, Bamato J. Itota, Patricia J. Kooyman, Darija Susac, Rhiyaad Mohamed

**Affiliations:** ^1^ HySA/Catalysis Centre of Competence Catalysis Institute Department of Chemical Engineering University of Cape Town Cape Town 7701 South Africa; ^2^ Theory and Computation of Energy Materials (IET‐3) Institute of Energy Technologies Forschungszentrum Jülich GmbH 52425 Jülich Germany; ^3^ SARChI Chair Nanomaterials for Catalysis Catalysis Institute Department of Chemical Engineering University of Cape Town Cape Town 7701 South Africa

**Keywords:** catalyst stability, electrocatalyst synthesis, hydrothermal synthesis, nano‐crystalline iridium dioxide, oxygen evolution reaction, perchlorate fusion, water electrolysis

## Abstract

Iridium oxides are the state‐of‐the‐art oxygen evolution reaction (OER) electrocatalysts in proton‐exchange‐membrane water electrolyzers (PEMWEs), but their high cost and scarcity necessitate improved utilization. Crystalline rutile‐type iridium dioxide (IrO_2_) offers superior stability under acidic OER conditions compared to amorphous iridium oxide (IrO_x_). However, the higher synthesis temperatures required for crystalline phase formation result in lower OER activity due to the loss in active surface area. Herein, a novel perchlorate fusion–hydrothermal (PFHT) synthesis method to produce nano‐crystalline rutile‐type IrO_2_ with enhanced OER performance is presented. This low‐temperature approach involves calcination at a mild temperature (300 °C) in the presence of a strong oxidizing agent, sodium perchlorate (NaClO_4_), followed by hydrothermal treatment at 180 °C, yielding small (≈2 nm) rutile‐type IrO_2_ nanoparticles with high mass‐specific OER activity, achieving 95 A g_Ir_^−1^ at 1.525 *V*_RHE_ in ex situ glass‐cell testing. Most importantly, the catalyst displays superior stability under harsh accelerated stress test conditions compared to commercial iridium oxides. The exceptional activity of the catalyst is confirmed with in situ PEMWE single‐cell evaluations. This demonstrates that the PFHT synthesis method leverages the superior intrinsic properties of nano‐crystalline IrO_2_, effectively overcoming the typical trade‐offs between OER activity and catalyst stability.

## Graphical abstract

A novel perchlorate fusion–hydrothermal synthesis for nano‐crystalline IrO_2_, utilizing low‐temperature calcination in the presence of an oxidant followed by hydrothermal treatment, yields ≈2 nm‐sized particles composed of nano‐crystalline iridium dioxide. This system shows a unique combination of high activity and high stability for the oxygen evolution reaction.

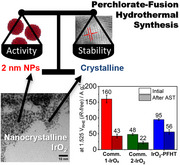

## 1 Introduction

Proton‐exchange‐membrane water electrolyzers (PEMWEs) provide an attractive solution for producing hydrogen from carbon‐neutral energy sources.^[^ ^1^ ^]^ However, the kinetically sluggish oxygen evolution reaction (OER) occurring at the anode causes severe energy losses, requiring the development of active and stable electrocatalysts.^[^ ^2^ ^]^ The strongly acidic and oxidizing anode environment restricts the choice of catalyst materials to the oxides of platinum‐group metals (PGM), with iridium oxides being the current state‐of‐the‐art.^[^ ^2^ ^]^ Due to the scarcity and cost of iridium, large‐scale deployment of PEMWE technologies could face an iridium supply bottleneck.^[^ ^3^ ^]^ To avoid this, anodic iridium loadings need to be reduced from 2.0 to less than 0.4 mg_Ir_ cm^−2^ without compromising the lifetime and performance of these catalysts.^[^ ^3^ ^]^

Previous efforts to enhance the utilization of iridium have largely focused on (hydrous) amorphous iridium oxides (IrO_x_)^[^ ^4^ ^]^ that are commonly considered to be more active towards the OER than crystalline rutile‐type iridium dioxide (IrO_2_).^[^ ^5^ ^]^ Amorphous IrO_x_ typically has a mixture of the formal Ir^3+/4+^ oxidation states and a significantly disordered structure. While amorphous IrO_x_ provides a higher Ir‐mass‐specific electrocatalytic activity, stability is often insufficient, thus jeopardizing the strategy for decreased iridium loading under long‐term operation in a PEMWE. Such an inverse correlation between catalyst activity and stability has widely been observed in OER electrocatalysis.^[^ ^6^ ^]^

Better stability under OER conditions is provided by crystalline IrO_2_,^[^ ^7^ ^]^ which is characterized by long‐range structural order and the predominance of the formal Ir^4+^ oxidation state.^[^ ^8^ ^]^ Recent experiments on single‐crystal IrO_2_ (110) films demonstrated impressive stability under extreme conditions at 2.1 V_SHE_ and a current density of 250 mA cm^−2^.^[^ ^9^ ^]^ The anomalous stability of crystalline IrO_2_ under OER conditions (“iridium dioxide anomaly”)^[^ ^10^ ^]^ can be explained by a particularly stable metal–oxygen (Ir─O) bond network in the IrO_2_ lattice.^[^ ^11^ ^]^ Whereas conventionally considered OER mechanisms require the cleavage of metal–oxygen bonds to release the oxygen molecule, a recently published computational study proposed a bi‐nuclear mechanism on the IrO_2_ (110) surface via an Ir*OOOO*Ir transition state.^[^ ^11^ ^]^ The novel mechanism thus provides an OER pathway that does not involve the breaking of Ir─O bonds, enabling the catalyst to be simultaneously active and stable, while defying the unfavorable, often‐observed activity–instability correlation.^[^ ^12^ ^]^ Crystalline IrO_2_ is therefore receiving increased attention in recent strategies towards PEMWE anode catalyst optimization.^[^ ^8^, ^13^ ^]^

Optimized synthesis routes for crystalline IrO_2_ nanoparticles (NPs) are required to leverage the exceptional combination of high intrinsic OER activity and stability.^[^ ^13^, ^14^ ^]^ Such efforts must be directed at increasing the specific surface area of crystalline IrO_2_, which typically has a much lower active surface area than amorphous IrO_x_. The formation of the IrO_2_ crystalline phase requires thermal treatment during conventional synthesis at high temperatures of at least 400 °C. This can be done via the oxidative heat treatment of a pre‐formed iridium metal,^[^ ^15^ ^]^ an amorphous IrO_x_ phase,^[^ ^4^, ^16^ ^]^ or as an inherent part of the synthesis process as in the case of Adams fusion^[^ ^17^ ^]^ or metal–organic chemical deposition (MOCD) methods.^[^ ^18^ ^]^ IrO_2_ crystallization at high temperatures coincides with particle growth, resulting in a loss of Ir‐mass‐specific surface area and decreased OER activity.^[^ ^4^, ^19^ ^]^ Thus, a trade‐off exists between crystallinity and active surface area for IrO_2_ obtained from calcination. To avoid unwanted particle growth, Malinovic et al. recently presented an approach to encapsulating iridium oxide NPs in silica nanoreactor shells during heat treatment.^[^ ^13b^ ^]^ With this method, the authors obtained IrO_2_ nanoparticles below 10 nm in size at calcination temperatures up to 800 °C. However, the complexity of the encapsulation method would most probably hamper commercial scale‐up.

As an alternative strategy, particle growth can be minimized by synthesis routes that yield crystalline IrO_2_ NPs at the lowest possible temperatures. Typical wet‐chemistry routes, including polyol^[^ ^20^ ^]^ and chemical reduction,^[^ ^21^ ^]^ are not suitable for this purpose, because they produce metallic iridium NPs and require subsequent calcination at high temperatures in an oxidizing atmosphere to obtain crystalline IrO_2_ as a final product. These methods utilize iridium chloride precursors, such as X_2_IrCl_6_ (X = H^+^, Na^+^, K^+^), with the iridium cation in a formal 4+ oxidation state, which is reduced in an alkaline environment.^[^ ^22^ ^]^ For a direct synthesis of crystalline IrO_2_, a promising strategy consists of adding a strong oxidant to avoid the reduction of the precursor, thereby minimizing the need for oxidative thermal treatment. Strong oxidants such as potassium superoxide (KO_2_) have previously been used to produce IrO_x_ colloids.^[^ ^23^ ^]^ The Adams fusion reaction, using NaNO_3_ as an oxidant, has gained significant attention for iridium oxide preparation.^[^ ^17^, ^24^ ^]^ It proceeds via a thermal melt reaction of NaNO_3_ with the Ir‐chloride precursor, where an iridium nitrate intermediate is formed which subsequently decomposes to yield crystalline IrO_2_. This method generally requires high temperatures of ≈500 °C, resulting in larger nanoparticles (>5 nm) and lower performance compared to amorphous iridium oxides.^[^ ^19^, ^24^ ^]^ Lower temperatures of 350 °C using iridium acetylacetonate (Ir(acac)_3_) as the precursor have been used for this method, leading to smaller particles.^[^ ^19b^ ^]^ However, the low vaporization temperature of this precursor resulted in low yields.^[^ ^18^ ^]^ Using H_2_IrCl_6_ as a precursor, Felix et al.^[^ ^24b^ ^]^ investigated the influence of synthesis temperature on the Adams fusion method. They concluded that treatment at 350 °C for 2 h yielded maximal OER activity of the obtained IrO_2_ electrocatalyst, while a lower synthesis temperature of 250 °C resulted in poor activity.

As an alternative, hydrothermal synthesis methods have been employed for producing iridium oxides, where an aqueous precursor solution is heated to ≈200 °C in an autoclave, allowing for increased solubility and reactivity of the precursors. However, these iridium oxides were found to be rather amorphous, requiring calcination at temperatures of 400 and 500 °C to form crystalline IrO_2_ phases, which then have larger particle sizes of 3.4 nm and 13.7 nm, respectively.^[^ ^25^ ^]^

In order to obtain very small, high‐surface‐area, rutile‐type IrO_2_ NPs, we have designed a two‐step synthesis method. An Adams fusion‐type reaction is first performed at 300 °C using sodium perchlorate as the oxidizing agent, followed by hydrothermal treatment at 180 °C under autogenous pressure, resulting in the desired highly crystalline rutile‐type IrO_2_ NPs of ≈2 nm. This “perchlorate fusion–hydrothermal (PFHT) synthesis” enables the combination of the stable crystalline nature of iridium dioxide with maximized iridium utilization due to the high surface‐to‐volume ratio of the nano‐sized particles, yielding a highly active, unsupported iridium dioxide catalyst with exceptional stability for OER in acidic conditions.

## 2 Results and Discussion

### 2.1 Synthesis of the Iridium Dioxide Catalyst

**Figure 1:**
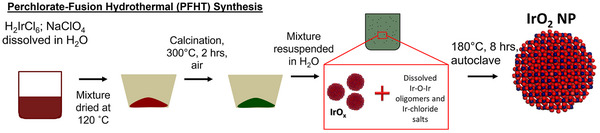
Schematic of the perchlorate fusion–hydrothermal (PFHT) synthesis.

The PFHT synthesis route is schematically shown in **Figure** **1**. The hexachloroiridate precursor (H_2_IrCl_6_.*x*H_2_O) and sodium perchlorate (NaClO_4_.*x*H_2_O) were dissolved in deionized water and dried at 120 °C. The dry mixture was calcined in static air at 300 °C for 2 h (heating rate 5 °C min^−1^) to yield a dark green hygroscopic crystalline solid. Upon redissolution/resuspension of this intermediate in water, two separate phases were observed: a dark green solution and a black solid component. The characterization of this two‐phase intermediate proved quite challenging; however, the black solid component was isolated and studied using transmission electron microscopy (TEM; Figure S1, Supporting Information), confirming that it consists of iridium oxide nuclei. The dark green solution is a mixture of dissolved iridium species; likely in the form of different Ir─O─Ir oligomers.^[^ ^16^, ^26^ ^]^ The initial calcination step yielded a mixture of these soluble oligomers, residual iridium chlorides, and IrO_x_ nuclei serving as seeds for subsequent IrO_2_ crystal growth.

To drive the controlled growth of crystalline IrO_2_ nanoparticles, the reaction intermediate was hydrothermally treated in an autoclave at 180 °C for 8 h under autogenous pressure.

To evaluate the influence of the oxidant, the synthesis was repeated without sodium perchlorate, leading to the formation of an undesired iridium metal phase in addition to IrO_2_ (see X‐ray diffraction (XRD) results in Figure S5, Supporting Information)_._ This indicates disproportionation of the precursor occurring in the absence of the oxidant. Sodium perchlorate thus acts as an oxidizing agent, maintaining the Ir^4+^ valency of the [IrCl_6_]^2−^ precursor and ensuring the direct formation of IrO_2_ without the formation of metallic iridium. Furthermore, omitting the initial calcination step and directly performing the hydrothermal treatment of the iridium precursor–perchlorate solution resulted in very low yields and was not pursued further. Initially, a small‐scale batch of ≈130 mg of catalyst was prepared, achieving a synthesis yield of ≈69% in terms of iridium. The method was scaled up to produce 5 g of the IrO_2_‐PFHT catalyst using a 1 L hydrothermal reactor. The yield obtained was 83% on an iridium basis, which we consider to be quite high, given that some syntheses result in only 30% Ir yield.^[^ ^27^ ^]^ The observed losses likely result from incomplete oxide formation. Nevertheless, the improved yield upon upscaling highlights the method's effectiveness for catalyst production. The physicochemical characterization presented refers to the small batch, whilst the electrochemical characterization (in situ and ex situ) was conducted on the upscaled batch. Notably, both batches exhibited similar structural and electrochemical properties, confirming the robustness and reproducibility of the PFHT synthesis methods (see Figures S3–S5, Supporting Information).

Energy‐dispersive X‐ray (EDX) analysis of the final IrO_2_‐PFHT catalyst confirmed the formation of iridium dioxide, with ≈72 wt.% Ir content, but also containing 5 wt.% chloride (Figure S2, Supporting Information). In principle, chlorides contained in the catalyst can be oxidized and contribute to the anodic currents recorded during OER activity evaluation. However, we do not consider this problematic, because the small chloride fraction would likely be electrochemically oxidized and evolved to chlorine gas within the first few seconds of the electrochemical protocol, given that IrO_2_ is an active catalyst towards the chlorine evolution reaction.^[^ ^28^ ^]^ As estimated in Section S1 (Supporting Information), even complete oxidation of the chloride fraction present in the prepared electrode layers would produce an oxidation charge of ≈2.7 mC only. This is negligible compared to typical OER current magnitudes of ≈1–10 mA generated during the activity testing, maintained over minutes or hours of the protocol. Therefore, any spurious contribution from chlorine evolution could have affected the recorded currents only during the very first seconds of the experimental protocol, thus excluding any possible bias of our OER activity data. However, to improve the yield of iridium dioxide in the final catalyst product, synthesis modifications, to reduce the chlorine content are underway as part of our current and future work.

### 2.2 Physical Characterization

**Figure 2:**
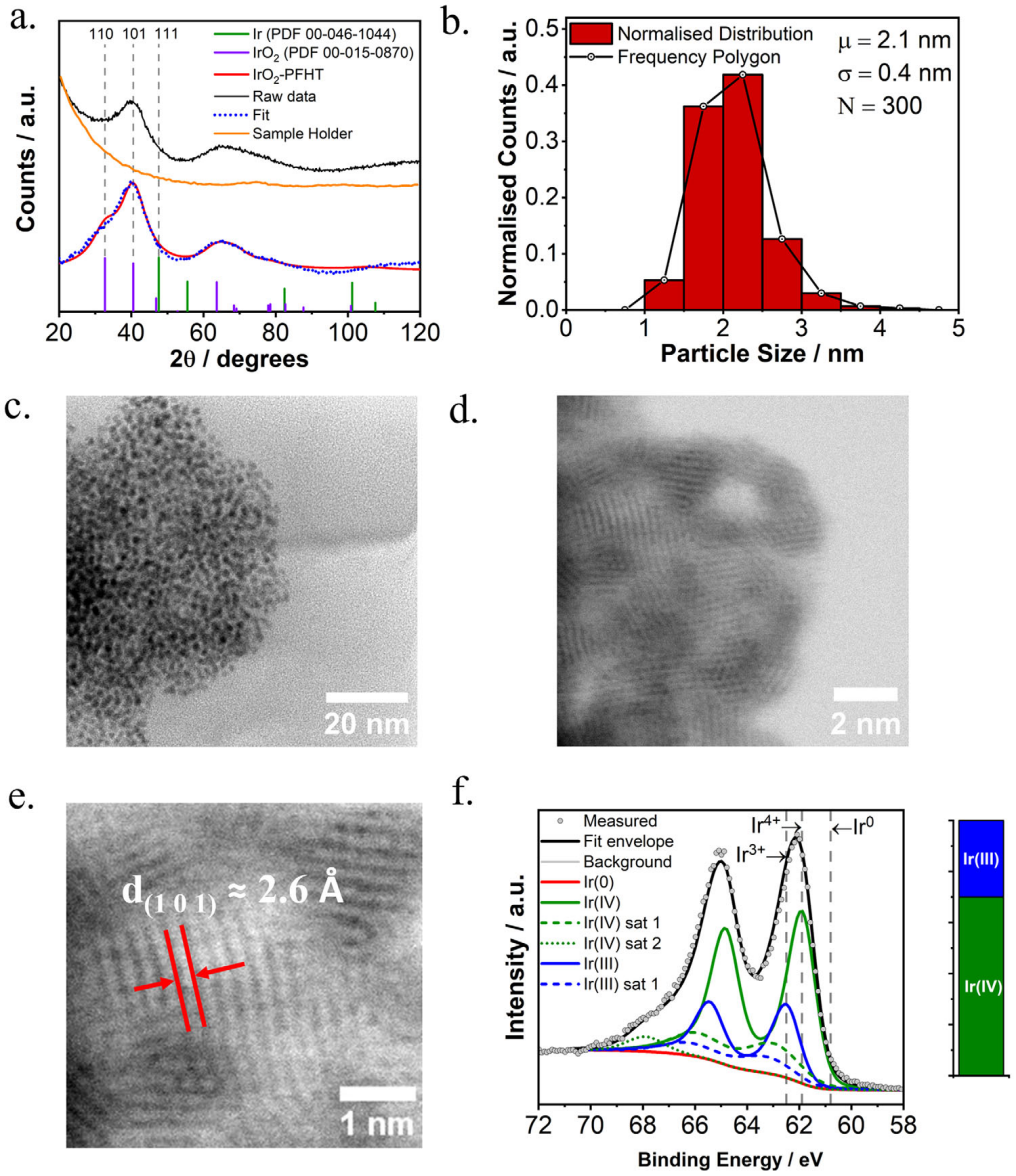
a) X‐ray diffractogram of the IrO_2_‐PFHT catalyst, corrected for the scattering background of the sample holder (red). The purple and green reference lines correspond to rutile‐type IrO_2_ (PDF 00‐015‐0870) and cubic Ir metal (PDF 00‐046‐1044), respectively. A fit with the Lorentzian‐broadened IrO_2_ reference peaks is shown, in good agreement with the pattern of the IrO_2_‐PFHT catalyst. b) Normalized particle size distribution and c,d) representative HRSTEM images of the IrO_2_‐PFHT catalyst. e) HRSTEM image showing the lattice fringes correlating to the (101) lattice plane of rutile IrO_2_. f) XPS peak fitting of the Ir 4f spectrum and the associated contributions of formal iridium valencies for the IrO_2_‐PFHT catalyst.

The X‐ray diffractogram of the IrO_2_‐PFHT catalyst, shown in **Figure** **2**a, reveals two broad diffraction lines indicating that the material consists of either a poorly crystallized material with short‐range order, or very small crystallites below the detection limit of the X‐ray diffractometer (<3 nm).^[^ ^29^ ^]^ The pattern is well reproduced by a fit with the Lorentzian‐broadened reference lines for rutile‐type iridium dioxide (PDF 00‐015‐0870),^[^ ^30^ ^]^ shown as a dashed curve, using variable relative intensities for the respective lines. According to the reference pattern for bulk IrO_2_, the reflection at 2θ  =  32.7° pertaining to the (110) lattice plane is expected to be the strongest reflection. However, for the IrO_2_‐PFHT material, the respective diffraction line is only visible as a shoulder of the dominant line at 2θ  =  40.5° corresponding to the (101) plane. We interpret this as result of a (110) in‐plane elongation of crystallites to preferentially expose the most stable (110) surface, resulting in a shortened extent of lattice planes in the respective out‐of‐plane direction. This behavior, termed “texturing”, has been observed in larger iridium dioxide NPs prepared using high‐temperature thermal treatment (>500 °C), where growth in the [001] direction results in domination of {110} surface terminations.^[^ ^19b^ ^]^ Our XRD result likely shows the initiation of this process and therefore displays an increased width of the (110) diffraction line at the expense of its height.  Furthermore, no reflexes pertaining to metallic iridium (PDF 00‐046‐1044)^[^ ^31^ ^]^ are visible in the XRD; indicating that no metallic iridium particles that exceed the 3 nm detection limit are present in the catalyst. This suggests that the use of the sodium perchlorate oxidant was successful in preventing the reduction or disproportionation of the iridium precursor (see also Figure S5, Supporting Information).

High‐resolution scanning transmission electron microscopy (HRSTEM) imaging was used to confirm the formation of nanoparticles and determine the particle size distribution (Figure 2b). As shown in Figure 2c,d, the IrO_2_‐PFHT catalyst is composed of small nanoparticles with an average particle size of 2.1 ± 0.4 nm. The crystalline nature of the particles is confirmed by the clearly visible lattice planes, as shown in Figure 2d,e. The measured d‐spacing of ≈2.6 Å is consistent with the reference value of 2.582 Å for the IrO_2_ (101) plane. Several particles with visible lattice fringes were measured to quantify the respective d‐spacings, a summary of which is presented in **Table** **1**. The measured d‐spacings are compared to the closest matching d‐spacing values of the rutile‐type IrO_2_ and cubic Ir metal lattices. All measured spacings are consistent with the rutile IrO_2_ lattice, but a few of the values would agree with both rutile‐type IrO_2_ and cubic Ir metal. Combined with the results from XRD, however, any relevant presence of an Ir metal phase can be excluded.

**Table 1:** Lattice spacings measured from TEM images of the IrO_2_‐PFHT catalyst for 12 particles and their comparison to the closest matching IrO_2_ and Ir metal hkl planes (see Figure S6, Supporting Information for the respective TEM images of the particles).

Particle	Measured d‐spacing/Å	Closest *d*‐spacing in tetragonal IrO_2_/Å	Closest *d*‐spacing in cubic Ir metal/Å	Most likely phase
1	2.02 ± 0.06	2.01 (210)	1.92 (200)	IrO_2_
2	2.26 ± 0.06	2.25 (200)	2.22 (111)	IrO_2_ or Ir
3	2.05 ± 0.06	2.01 (210)	2.22 (111)	IrO_2_
4	2.27 ± 0.06	2.25 (200)	2.22 (111)	IrO_2_ or Ir
5	2.30 ± 0.06	2.25 (200)	2.22 (111)	IrO_2_ or Ir
6	2.27 ± 0.06	2.25 (200)	2.22 (111)	IrO_2_ or Ir
7	2.26 ± 0.06	2.25 (200)	2.22 (111)	IrO_2_ or Ir
8	2.63 ± 0.06	2.58 (101)	2.22 (111)	IrO_2_
9	2.64 ± 0.06	2.58 (101)	2.22 (111)	IrO_2_
10	2.62 ± 0.06	2.58 (101)	2.22 (111)	IrO_2_
11	2.61 ± 0.06	2.58 (101)	2.22 (111)	IrO_2_
12	2.63 ± 0.06	2.58 (101)	2.22 (111)	IrO_2_

X‐ray photoelectron spectroscopy (XPS) was employed to investigate the chemical nature of the prepared IrO_2_‐PFHT catalyst. The Ir 4f region of the XPS spectrum is shown in Figure 2f The Ir 4f_7/2_ reference binding energy for iridium metal is located at 60.8 ± 0.2 eV,^[^ ^32^ ^]^ whilst the Ir 4f_7/2_ reference binding energy for iridium in a formal 4+ oxidation state, as in IrO_2_, is found at a higher value of 61.9 ± 0.5 eV.^[^ ^32^ ^]^ Peak fitting of the high‐resolution Ir 4f spectrum was carried out to quantify the proportions of different iridium oxidation states contributing to the Ir 4f spectrum. The catalyst exhibits predominantly Ir^4+^ character, with the remaining contribution from Ir^3+^ potentially due to the presence of surface oxohydroxide species or adsorbed Ir─Cl species.^[^ ^8a^ ^]^ No Ir^0^ (metallic iridium) component was detected.

Physical characterization by XRD, HRSTEM, and XPS thus consistently confirms the highly crystalline nature of the synthesized IrO_2_ nanoparticles. While the presence of an amorphous IrO_x_ cannot be entirely excluded, the data presented here demonstrate that nano‐crystalline IrO₂ is the dominant phase.

### 2.3 Ex Situ Electrochemical Characterization

**Figure 3:**
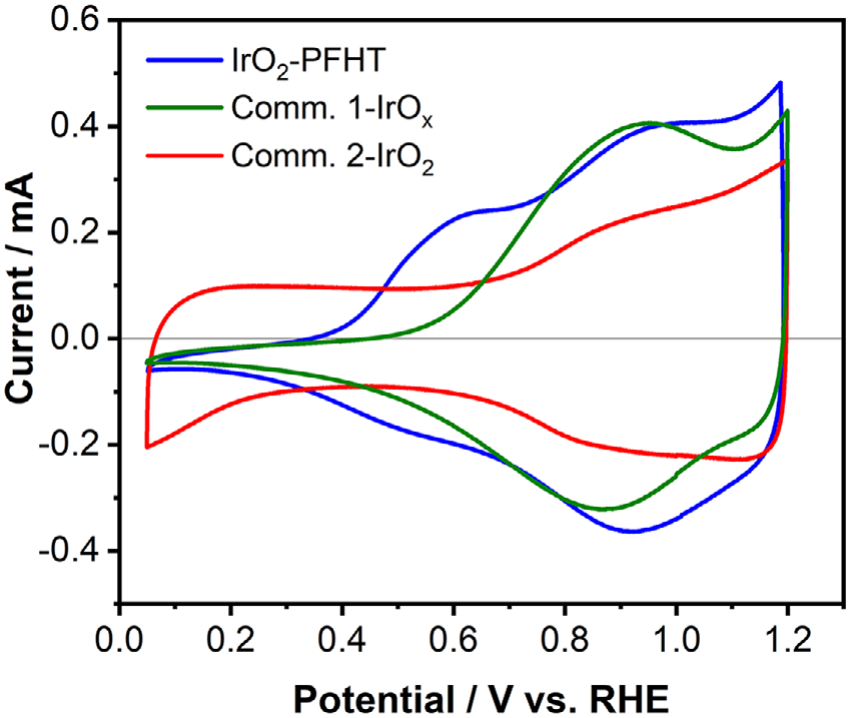
Cyclic voltammogram (CV) of the IrO_2_‐PFHT catalyst, recorded at a sweep rate of 50 mV sec^−1^ mV/s between 0.050–1.200 V versus RHE in 0.5 м H_2_SO_4_ solution, in comparison to the commercial benchmark iridium oxides. Comm. 1‐IrO_x_ is a highly active Ir‐based catalyst, whilst Comm. 2‐IrO_2_ catalyst is a crystalline iridium oxide selected for its inherent stability. The OER activity and stability of the prepared catalyst were investigated ex situ in a three‐electrode glass‐cell setup in 0.5 м H_2_SO_4_ electrolyte.

**Figure** **3** displays the cyclic voltammogram (CV) of the IrO_2_‐PFHT catalyst in 0.5 м H_2_SO_4_ electrolyte recorded in the potential range of 0.050–1.200 V versus RHE (blue curve), compared against two commercial benchmark catalysts: a highly active Ir‐based catalyst, labeled Comm. 1‐IrO_x_, and a highly crystalline catalyst(Comm. 2‐IrO_2_) selected for its inherent stability. The CV of the IrO_2_‐PFHT catalyst is characterized by a large pseudocapacitance at potentials above ≈0.900 V versus RHE, which can be attributed to the de‐/protonation of bridging oxygen sites at the IrO_2_ surface.^[^ ^11^ ^]^ In the potential range below ≈0.5 V versus RHE, the apparent capacitance is drastically suppressed, which is also observed for the commercial Comm.1‐IrO_x_ catalyst (green curve in Figure 3). This phenomenon is commonly attributed to the bulk insertion of protons,^[^ ^33^ ^]^ effectively reducing the iridium oxide to an iridium oxyhydroxide (IrOOH) with a formal Ir^3+^ valency. Since Ir^3+^ corresponds to an electronic 5d^6^ configuration with a fully occupied t_2_ _g_ sub‐band,^[^ ^34^ ^]^ the bulk‐reduced IrOOH is an insulator with a very low electrochemical response, explaining the suppressed capacitance at low potentials. In contrast, the pristine bulk IrO_2_ phase with a formal Ir^4+^ valency, prevalent at high potentials, has metallic conductivity.^[^ ^33a^ ^]^ The electrochemical switching between the conductive high‐potential and the insulating low‐potential states is commonly considered as a signature of amorphous iridium oxides due to the facile insertion of protons into the amorphous bulk structure. In contrast, crystalline IrO_2_ electrodes such as the commercial Comm. 2‐IrO_2_ benchmark (red curve in Figure 3), do not typically exhibit such switching behavior because the crystalline bulk structure does not allow for sufficient proton mobility. Here, we observe that despite its high crystallinity, the IrO_2_‐PFHT catalyst displays electrochemical bulk reduction, albeit occurring at slightly more negative potentials in comparison to the amorphous Comm.1‐IrO_x_ benchmark. We consider the susceptibility of the IrO_2_‐PFHT catalyst to bulk reduction to be a consequence of its nano‐crystalline nature. The very small particle size of the IrO_2_‐PFHT catalyst could allow for protonation of most of the particle volume despite the slow proton mobility in the crystalline IrO_2_ structure. Temperature‐programmed reduction (TPR) experiments were performed on the IrO_2_‐PFHT catalyst to gain additional insights into its reducibility behavior. As shown in Figure S7a,b of the Supporting Information, the TPR results indicate that the synthesized catalyst is much easier to reduce than commercial Comm. 2‐IrO_2_. Furthermore, it is confirmed that IrO_2_‐PFHT was fully reduced during TPR, as evidenced by the XRD of the post‐TPR product (Figure S7c, Supporting Information) lacking any signal for iridium dioxide. These results corroborate the CVs shown in Figure 3 and confirm that the nano‐crystalline character of the IrO_2_‐PFHT catalyst resulted in enhanced chemical and electrochemical reducibility.

**Figure 4:**
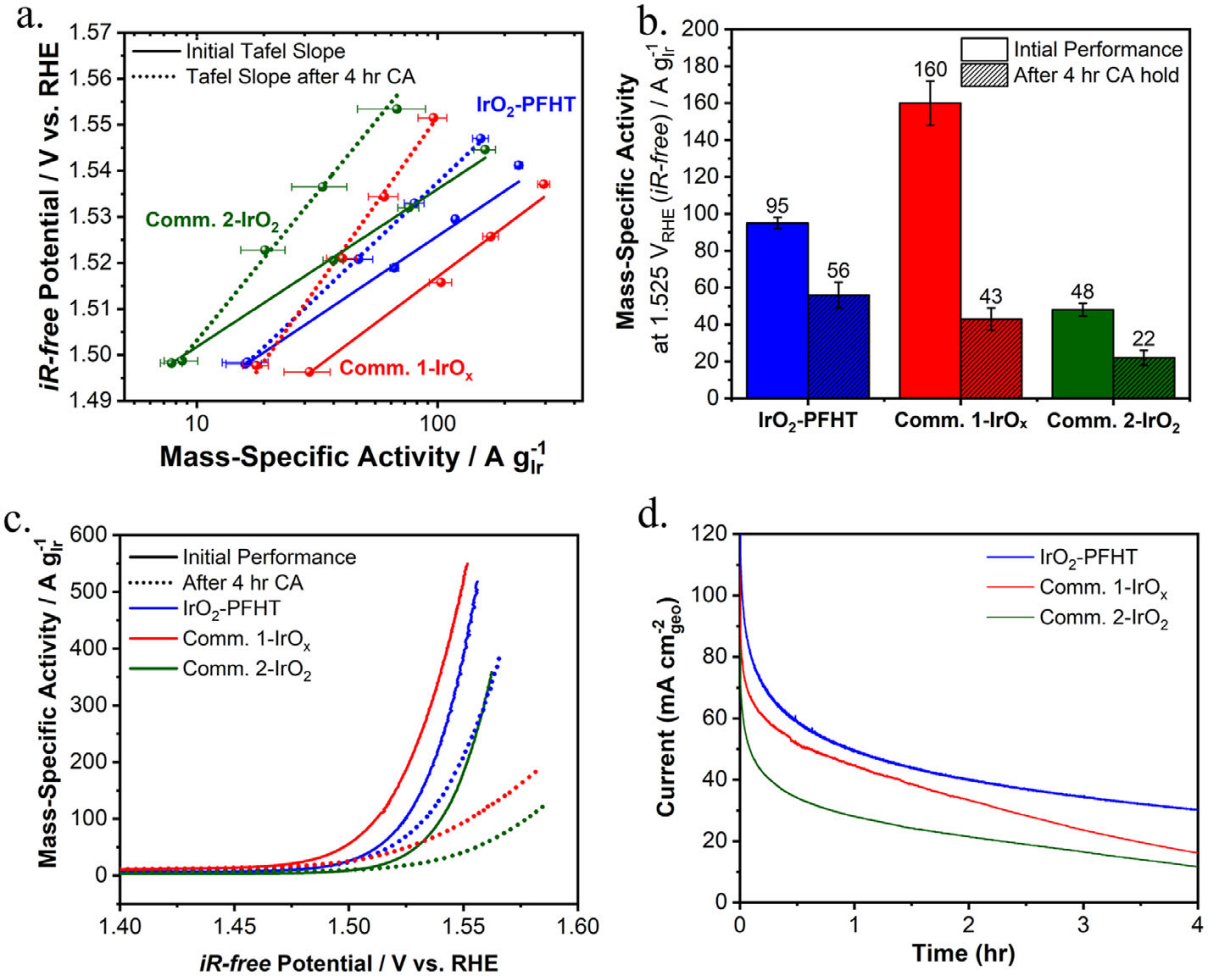
a) Tafel plots of the OER currents for the IrO_2_‐PFHT catalyst, commercial IrO_x_, and IrO_2_ benchmark catalysts (labeled Comm.1 and Comm. 2, respectively) before (solid lines) and after (dotted lines) the AST stability evaluation. b) Ir‐mass‐specific current densities at 1.525 V_RHE_ before and after the AST. c) Current‐potential curves of the IrO_2_‐PFHT catalyst and the commercial IrO_x_ and IrO_2_ benchmarks before and after the AST. The curves shown are averaged forward and backward cyclic voltammetry sweeps measured from 1.400–1.600 V_RHE_ at a scan rate of 10 mV dec^−1^. d) AST chronoamperometry at 1.600 V_RHE_ for 4 h including online *iR*‐correction accounting for 85% of the Ohmic drop. The RDE disc was rotated at 2000 rpm during the AST (catalyst loading on the electrode: 100 µg_cat_ cm_geo_^−2^; electrolyte: 0.5 м H_2_SO_4_).

Tafel plots of the Ir‐based mass‐specific OER currents recorded before and after the accelerated stress test (AST) are shown in **Figure** **4**a. Figure 4b compares the Ir‐based mass‐specific current density at 1.525 V versus RHE, obtained by linear interpolation of the Tafel slopes, for the investigated catalysts. The initial activity of the IrO_2_‐PFHT catalyst was 95(±3) A g_Ir_^−1^ at 1.525 V versus RHE, an excellent result for a crystalline iridium dioxide catalyst. In particular, the IrO_2_‐PFHT catalyst was twice as active as the commercial IrO_2_ (Comm. 2‐IrO_2_) catalyst. On the other hand, the IrO_x_ benchmark (Comm. 1‐IrO_x_) demonstrated ≈70% higher initial activity compared to the Comm. 2‐IrO_2_, as expected for an amorphous iridium oxide catalyst. All three catalysts revealed very similar initial Tafel slopes with a value of 35 mV dec^−1^ for the IrO_2_‐PFHT catalyst and 39 and 34 mV dec^−1^ for the Comm. 1‐IrO_x_ and Comm. 2‐IrO_2_ commercial benchmarks_,_ respectively. Our results thus demonstrate consistently low Tafel slopes for the crystalline and amorphous iridium oxide commercial catalysts. While the quantitative activity comparison was based on chronoamperometric step experiments with a one‐minute holding at each potential (cf. Experimental Methods), current‐potential curves are presented in Figure 4c for a more direct visual comparison of the catalysts.

**Figure 5:**
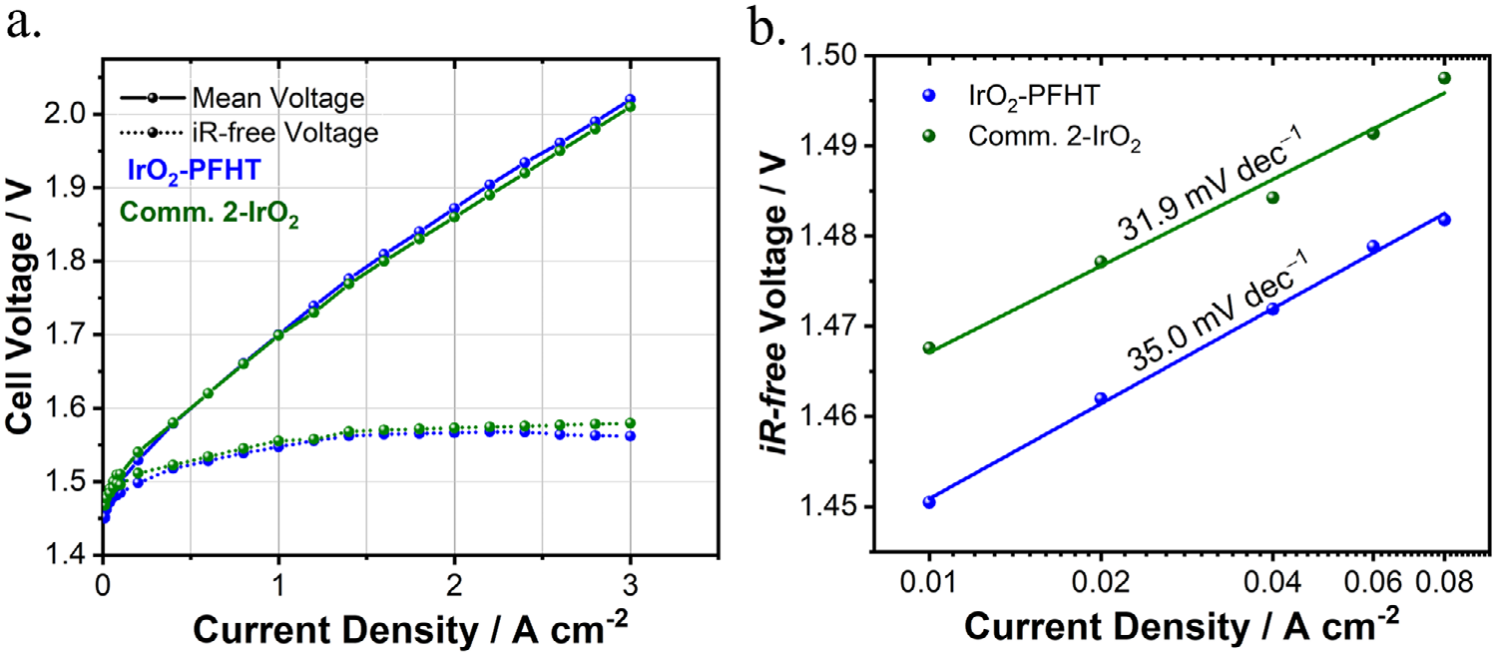
In situ, single‐cell PEM water electrolysis performance of CCMs consisting of IrO_2_‐PFHT (blue) and Comm. 2‐IrO_2_ catalyst (green) as the anodic catalysts. a) Ambient pressure polarization curves obtained at 60 °C b) Tafel plots constructed from the kinetic region (0.01–0.08 A cm^−2^) of the polarization curves.

To assess the long‐term stability, the catalysts were subjected to an AST consisting of a 4 h chronoamperometric experiment at 1.6 V versus RHE, followed by a repeat of the activity protocol. While galvanostatic AST protocols are more commonly used,^[^ ^4^, ^35^ ^]^ we consider chronoamperometry more suitable for a fair comparison of intrinsic catalyst stability. Since OER catalyst degradation is a potential‐driven process, stability testing at a constant potential allows for fair comparison between different catalyst materials.^[^ ^36^ ^]^ However, AST protocols based on chronoamperometry can result in significant differences between the applied versus actual potential that is experienced by a given catalyst. To account for this discrepancy, careful treatment of Ohmic losses is required. Without *iR*‐correction, the effective potential to which the catalyst is exposed is much lower than the applied potential. This is further exacerbated in more active catalysts that produce higher currents, resulting in a greater *iR*‐drop. To account for this, an automatic/online *iR*‐correction was applied during chronoamperometry at 1.600 V_RHE_, ensuring that each catalyst was subjected to consistently strenuous corrosive conditions (Figure S7, Supporting Information). To prevent potentiostatic oscillations or over‐compensation, only 85% of the Ohmic resistance (determined by electrochemical impedance spectroscopy (EIS) prior to the AST) could be used for online correction. Additionally, experiments were conducted in 0.5 m H₂SO₄, as in this electrolyte a significantly lower solution resistance (5–7 Ω) is obtained, compared to the usage of 0.1 m HClO₄ electrolyte where Ohmic resistances in the range of 25–30 Ω are typically observed. The lower electrolyte resistance of H_2_SO_4_ minimizes the Ohmic drop, ensuring more accurate potential control during stability measurements. The remaining 15% thus corresponded to a negligible uncorrected resistance of ≈1 Ω. We finally note that the effective potential of 1.600 V versus RHE to which catalysts were exposed during this AST protocol corresponds to harsh corrosive conditions and is representative of the potentials experienced during in situ PEM electrolyzer testing, where the iR‐free potential typically does not exceed 1.6 V as demonstrated by the iR‐free voltage curve in **Figure** **5**a.

Comparing the Ir‐mass‐specific activities before and after the AST, cf. Figure 4b, the IrO_2_‐PFHT catalyst is found to be exceptionally stable. Whilst the IrO_x_ benchmark (Comm. 1‐IrO_x_) catalyst lost ≈73% of its initial performance, the activity of the IrO_2_‐PFHT catalyst decreased by only 41%, even less than the decrease observed for the (significantly less active) commercial IrO_2_ benchmark (Comm. 2‐IrO_2_) that lost 54% of its initial activity. As a result, after the AST, the IrO_2_‐PFHT catalyst was the most active among the investigated catalysts, achieving a remarkable 56 (±7) A g_Ir_^−1^ at 1.525 V_RHE_. Inspecting the chronoamperometric curves recorded during AST, shown in Figure 4d, the IrO_2_‐PFHT and Comm. 2‐IrO_2_ catalysts demonstrate similar degradation rates despite the higher current density achieved by the former, whilst the commercial Comm.1‐IrO_x_ benchmark reveals a much steeper degradation slope over the 4 h period. Therefore, extending the period of the AST protocol would have likely further enhanced the observed trends in end‐of‐test (EOT) activities between the catalysts. Catalyst degradation has been shown to take place through the dissolution of iridium,^[^ ^7^ ^]^ detachment of catalyst particles from the substrate,^[^ ^37^ ^]^ and particle growth.^[^ ^37^ ^]^ Stability evaluations using the RDE setup can also give rise to perceived degradation through the passivation of the RDE electrode substrate,^[^ ^38^ ^]^ or from the accumulation of nano‐ and micro‐oxygen bubbles in the catalyst layer.^[^ ^39^ ^]^ No detachment of the catalyst layer from the glassy carbon electrode was visually observed during any of the ASTs, and to aid oxygen bubble removal, the working electrode was rotated at 2000 rpm during the stability test. We therefore consider the observed activity degradation to be representative of either Ir dissolution or particle agglomeration/growth.

Tafel slopes obtained before and after the ASTs are summarized in **Table** **2**. For all three catalysts, the respective Tafel slope values increased after stability testing; however, the IrO_2_‐PFHT catalyst had the lowest EOT Tafel slope, which was consistent with its highest EOT mass‐specific activity. The value of the apparent Tafel slope can be influenced by many factors such as catalyst loading on the electrode, the pH of the electrolyte, and the electrochemical measurement protocol.^[^ ^40^ ^]^ However, since all measurements were conducted under identical conditions, we can infer that the changes in the Tafel slope reflect catalyst degradation, suggesting a change in the OER kinetics, likely due to structural or compositional modifications of the catalyst over time.

**Table 2:** Tafel slopes were determined before and after the ASTs.

Catalyst	Initial Tafel slope/mV dec^−1^	EOT Tafel slope/mV dec^−1^
IrO_2_‐PFHT	35 ± 1.5	50 ± 0.6
Comm. 1‐IrO_x_	39 ± 2.1	73 ± 3.5
Comm. 2‐IrO_2_	34 ± 0.7	64 ± 2.9

In **Table** **3**, the performance of our IrO_2_‐PFHT catalyst is compared to other unsupported iridium oxides synthesized via high‐temperature (350–450 °C) thermal treatments. The IrO_2_‐PFHT catalyst outperforms all catalysts prepared using the Adams fusion method. One such catalyst, synthesized at 350 °C using Ir(acac)_3_, was chosen to mitigate residual chlorine in the sample.^[^ ^19b^ ^]^ However, despite this precaution, its activity remains significantly lower than the IrO_2_‐PFHT catalyst, which demonstrates a threefold improvement. TEM and XPS analyses indicate comparable particle size, crystallinity, and morphology, but the scalability of this modified Adams fusion method is questionable due to the considerably higher cost of the Ir(acac)_3_ precursor compared to iridium chloride precursors (H_2_IrCl_6_ or IrCl_3_).^[^ ^19b^ ^]^

**Table 3:** A comparison of the IrO_2_PFHT catalyst performance against other iridium oxide catalysts from literature.

Refs.	Synthesis method	Particle diameter/nm	Tafel slope/ mV dec^−1^	*iR*‐free Potential/V_RHE_	Ir‐mass‐specific activity/A g_Ir_^−1^	
					Ref.	This work
[24a]	Adams fusion, IrO_2_ nanoneedles 450 °C	2.0	57	1.55*	61†	490 (extrapolated)
[19b]	Adams fusion @ 350 °C	1.7	44	1.525	44	95
[13b]	Sol‐gel silica encapsulation @ 400 °C	3.5	50	1.55	700‡	490 (extrapolated)

^*^ Potential not iR‐free

^†^ Mass‐specific activity converted to A g_Ir_^−1^ based on the theoretical Ir content of 86 wt.% for stoichiometric rutile‐type IrO_2_

^‡^ Mass‐specific activity given A g_oxide_^−1^

Adams fusion nanoneedles, synthesized at 450 °C using an iridium chloride precursor,^[^ ^24a^ ^]^ exhibited thermodynamically controlled growth, forming elongated particles (29.7 nm long, 2 nm wide). These are considerably larger than the 2.1 nm nanoparticles in our IrO_2_‐PFHT catalyst, indicating a greater surface area per mass for our material. The nanoparticles prepared via the same method without the cysteamine formed 2.9 nm‐sized unshaped particles which exhibited much lower performance.

Malinovic et al. encapsulated IrO₂ nanoparticles (3.5–7 nm) in a silica matrix to form an IrO₂ core – SiO₂ shell structure.^[^ ^13b^ ^]^ The sample prepared at 400 °C with 3.5 nm particles achieved 700 A g_oxide_⁻¹ at 1.55 V_RHE_, comparable to our catalyst's performance of 490 A g_Ir_⁻¹. However, this synthesis is complex, requiring a reverse‐microemulsion method, multiple reagents and surfactants, and hydrofluoric acid for silica removal — posing significant challenges for industrial‐scale production.

Overall, our catalyst demonstrates remarkable activity compared to these alternatives, making it a strong candidate for further development in electrolyzer applications.

### 2.4 In Situ Electrochemical Characterization

The IrO_2_‐PFHT catalyst demonstrates promising electrochemical performance towards the OER under ex situ conditions. However, the intrinsic electrochemical properties of electrocatalysts do not necessarily guarantee favorable in situ performance when integrated into industrially relevant PEM water electrolysis stacks and systems. In an electrolyzer, the catalyst is part of a more complex and dynamic membrane electrode assembly (MEA), where various components and their interactions can significantly influence the overall in situ performance of these devices. Therefore, evaluating the performance of novel materials under realistic working conditions is critical to the research and development of technologically relevant electrocatalysts.^[^ ^41^ ^]^

To evaluate the in situ electrochemical performance of the IrO_2_‐PFHT catalyst, catalyst‐coated membranes (CCMs) were fabricated using ultrasonic spray‐coating and tested in single‐cell PEM electrolyzer configuration. Steady‐state polarization curves measured at 60 °C from CCMs consisting of the IrO_2_‐PFHT and the Comm. 2‐IrO_2_ catalysts are shown in **Figure** **5**a. In both MEAs, all known cell components were identical; including the membrane, cathode composition, and cathodic and anodic porous transport layers (PTLs) used. The only variation was in the anode catalyst material and composition (i.e., ionomer content), allowing for an investigation of their respective activity in the MEA configuration. Both MEAs demonstrated similar beginning‐of‐life (BoL) performance up to a current density of 3.0 A cm^−1^ without noticeable mass transport losses, as shown in Figure 5a. The BoL performance aligns well with the U.S. Department of Energy's (DoE) target for in situ electrolyzer performance, which specifies that with a combined PGM loading of 3.0 mg_PGM_ cm^−2^, the cell voltage at 2.0 A cm^−2^ should not exceed 1.9 V.^[^ ^42^ ^]^ Our CCMs achieved a voltage of 1.87 V and 1.86 V at 2 A cm^−2^ for IrO_2_‐PFHT and Comm. 2‐IrO_2_, respectively.

Slight differences were observed in the kinetic region, where voltages of 1.45 V and 1.47 V were attained at a low current density of 0.01 A cm^−2^ for the IrO_2_‐PFHT and Comm. 2‐IrO_2_ CCMs, respectively. Tafel slopes of 35.0 and 31.9 mV dec^−1^ were obtained from the 0.01–0.08 A cm^−2^ region for the IrO_2_‐PFHT and commercial IrO_2_ CCMs (Figure 5b). These Tafel slopes are comparable to those reported for CCMs of similar loading and composition on N115 membrane,^[^ ^43^ ^]^ and agree well with the Tafel slopes attained from the ex situ electrochemical characterization. Furthermore, the ex situ and in situ results have shown to be consistent, with a similar relationship between the performance of IrO_2_‐PFHT and Comm. 2‐IrO_2_ being observed. For example, at a potential of 1.48 V (*iR‐free*), the IrO_2_‐PFHT catalyst outperformed the Comm. 2‐IrO_2_ catalyst by a factor of ≈2.5 in the glass cell whilst in the CCM, the current density obtained at this potential with the IrO_2_‐PFHT catalyst was 3 times that of the Comm. 2‐IrO_2_ catalyst.

EIS spectra and high‐frequency resistance (HFR)‐density plots are illustrated in Figure S9 (Supporting Information) with mean HFR values of 153 ± 3.5 mΩ cm^2^ and 143 ± 3.5 mΩ cm^2^ for IrO_2_‐PFHT and Comm. 2‐IrO_2_, respectively. The HFR decreased slightly with increasing current density (Figure S9a, Supporting Information), possibly due to a local increase in temperature.^[^ ^44^ ^]^

It should be noted that CCMs were prepared for the Comm. 1‐IrO_x_ catalyst as well; however, due to high electrical contact resistance between the anode catalyst layer and the porous transport layer (PTL), it was not possible to measure polarization curves. The HFR versus current density profile for this material can be found in Figure S10 of the Supporting Information.

## 3 Conclusion

In summary, a novel PFHT method for the synthesis of nano‐crystalline iridium dioxide catalysts for the OER was developed. The results from ex situ electrochemical characterization demonstrate an outstanding combination of OER activity and stability provided by the IrO_2_‐PFHT catalyst. The initial activity of IrO_2_‐PFHT was only slightly lower than that of the highly active commercial IrO_x_ benchmark (Comm. 1‐IrO_x_), but superior to the IrO_2_ benchmark (Comm. 2‐IrO_2_). Most importantly, the IrO_2_‐PFHT catalyst was significantly more stable than both commercial benchmarks, with only a moderate loss in activity after a rigorous stability evaluation. Accordingly, after the AST, the IrO_2_‐PFHT catalyst shows great potential for durable long‐term electrolyzer operation.

This promising performance seems to be due to the presence of very small IrO_2_ nanoparticles. X‐ray diffraction showed broad reflexes that were due to the very small (≈2 nm) crystalline IrO_2_ nanoparticles observed using HRSTEM. The strategy of decreasing the required synthesis temperature by employing a strong sodium perchlorate oxidant in a hydrothermal treatment thus successfully leveraged the activity–stability combination provided by nano‐crystalline IrO_2_ particles.^[^ ^11^, ^12^ ^]^

The reproducibility and scalability of the PFHT method were demonstrated by two different synthesis batches, with one of them successfully scaled up to the 5 g batch size, whilst maintaining its advantageous physiochemical and electrochemical properties. The PFHT synthesis approach thus appears highly promising to be pursued toward scaling up catalyst production.

Finally, we have demonstrated the application of the IrO_2_‐PFHT catalyst in a CCM, under in situ PEM electrolysis working conditions. The catalyst maintained its outstanding electrochemical performance and exhibited BoL performance in accordance with U.S. DoE targets. The focus of our current and future work involves further investigation of this material, with emphasis on its stability under in situ electrolyzer conditions, including characterization of the catalyst layer at the beginning and end of life to assess catalyst degradation.

## 4 Experimental Section

### Catalyst Synthesis

For the PFHT synthesis method, 240 mg (0.590 mmol) of dihydrogen hexachloroiridate hydrate, H_2_IrCl_6_.*x*H_2_O precursor (Sigma Aldrich, >99.9% trace metal basis), and 655 mg (5.35 mmol) of NaClO_4_.*x*H_2_O (Kimix chemicals) were dissolved in a minimal (≈20 mL) volume of deionized water in a crucible. The mixture was dried at 120 °C. The salt mixture was then calcined in a muffle furnace (Labofurn) for 2 h at 300 °C (heating rate 5 °C min^−1^) and allowed to cool to room temperature. A dark green hygroscopic solid was obtained and redispersed in 40 mL of deionized water. 10 mL aliquots of this mixture were transferred into four 30 mL PTFE‐lined steel autoclaves (Anton Parr) and placed in a muffle furnace for 8 h at 180 °C. The resultant black powder was separated from the clear solution and washed several times with deionized water using centrifugation at 7000 rpm for 10 min. The upscaled 5 g batch was prepared following the same procedure with 9.16 g (22.5 mmol) of the H_2_IrCl_6_.*x*H_2_O precursor and 22.3 g (0.18 mol) of NaClO_4_.*x*H_2_O dissolved in 40 mL of water. The intermediate was resuspended in 750 mL of water and hydrothermally treated in a customized PTFE‐lined 1 L hydrothermal reactor (Berghof BR‐1000) with stirring at 150 rpm. The resultant black powder was washed and separated from the supernatant via centrifugation at 7000 rpm for 10 min.

### Transmission Electron Microscopy (TEM)

A Tecnai F20 TEM operated at 200 kV with a field emission gun was used for the collection of bright field images.

### High‐Resolution Scanning Transmission Electron Microscopy (HRSTEM)

Images were recorded using a JEOL JEM ARM200F double Cs‐corrected electron microscope equipped with a field emission gun (FEG). At least 300 particles were measured to plot the particle size distribution histogram in which the maximum diameter of each particle was measured using the ImageJ software. *d*‐Spacings were measured by creating Fast Fourier Transforms (FFT) of the particles of interest, selecting the diffraction spots in the FFT image, and generating the inverse FFT. The d‐spacings were then measured with the plot profile option in Image J.

### X‐ray Diffraction (XRD)

Powder X‐ray diffractograms were obtained using a Bruker D8 Advance diffractometer with a Co‐K_a_ source (λ= 1.78897 Å). Powder samples were scanned in a 2Θ range of 20–120° with a 2Θ step size of 0.009° and a dwell time of 0.51 s per step. For further analysis, the data were averaged over bins of 20 raw data points, yielding one averaged data point every 0.18° in 2Θ. Each powder sample was ground with a mortar and pestle to homogenize the sample and remove any preferential alignment of the particles. Furthermore, the sample was loaded onto a zero‐background sample holder to avoid any interference from diffraction peaks originating from the sample holder.

### X‐ray Photoelectron Spectroscopy (XPS)

XPS measurements were performed at Rutgers K‐Alpha XPS facility using a Thermo Scientific K‐Alpha Photoelectron Spectrometer equipped with a monochromated, low‐power Al K α (1486.6 eV) X‐ray source. XPSpeak 4.1 software was used for data analysis, and peak fitting of the Ir 4f spectrum was carried out in accordance with literature fitting parameters.^[^ ^18^, ^32^ ^]^ The background‐corrected data were fitted using a full width at half maximum (FWHM) of 1.2 eV for the Ir^4+^ and Ir^3+^ peaks located at 61.9 eV (Ir4f_7/2_) and 62.5 eV (Ir4f_7/2_), respectively, 2.4 eV for the Ir^4+^ and Ir^3+^ satellite peaks located at 63.0 eV and 63.3 eV respectively, and 1.7 eV for the Ir^4+^ second satellite peak situated at 67.9 eV. The Gaussian‐Lorentzian ratio was set to 80:20 for all fits. The asymmetry factor (TS) and asymmetric tailing factor (TL) were set to 0.2 and 100, respectively, for the Ir^3+^, and Ir^4+^ peaks, whilst for the Ir^3+^ and Ir^4+^ satellite peaks, 0 and 1 were used for the TS and TL factors, respectively.

### Energy‐dispersive X‐ray (EDX)

EDX measurements were carried out using an FEI Nova Nano SEM 230 equipped with a field emission gun and an Oxford X‐max detector operated at 20 keV at a magnification of 1000 times with 300 × 300 µm sized squares to collect spectra.

### Temperature‐programmed Reduction (TPR)

The H_2_‐TPR experiments were performed using a Micromeritics Autochem 2950 instrument (Micromeritics, Atlanta, GA, USA). 50.0 mg of each catalyst was loaded into a quartz U‐tube reactor, embedded between two layers of quartz wool to ensure uniform heat and mass transfer through the catalyst bed. The samples were degassed in argon by heating to 120 °C over 3 h to remove any physisorbed substances from the catalysts before reduction. After the samples cooled to room temperature, the gas atmosphere was changed to a mixture of 5% H_2_ in Ar at a flow rate of 50 mL min^−1^. The temperature of the catalyst bed was ramped up to 600 °C at a rate of 10 °C min^−1^. Using a thermal conductivity detector, the H_2_ consumed by the catalyst was measured by comparing the difference between the incoming H_2_‐Ar gas mixture and the effluent gas.

### Ex Situ Electrochemical Characterization—Three‐Electrode Glass Cell Set‐up

The electrochemical characterization of the prepared catalysts towards the oxygen evolution reaction (OER) was performed ex situ in a three‐electrode glass cell at room temperature using a glassy‐carbon rotating disc electrode (RDE) as the substrate for the catalyst layer. Prior to all electrochemical measurements, the glassware was cleaned of organic and inorganic contaminants by first soaking the glassware in a 1:1 v:v H_2_SO_4_:H_2_O solution for 24 h and thoroughly rinsing with ultrapure (18 MΩ Merck Millipore) deionized water before boiling in water overnight to remove all residual sulfuric acid. The setup consisted of a three‐electrode glass cell (150 mL, Pine Research) filled with ≈100 mL of aqueous 0.5 м sulfuric acid (H_2_SO_4_) solution as the electrolyte (99.999%, Sigma Aldrich), through which oxygen gas was bubbled during OER measurements. The counter electrode was a platinum wire where the hydrogen evolution reaction (HER) served as a cathodic counter‐reaction for the anodic OER on the working electrode. Before each electrochemical experiment, the counter electrode was flame‐cleaned and rinsed with 18 MΩ Millipore deionized water. A Hg/Hg_2_SO_4_/K_2_SO_4_ (sat.) electrode served as the reference electrode. To report the applied potential with respect to the reversible hydrogen electrode (RHE), the reference electrode was calibrated prior to each electrochemical testing series by saturating the electrolyte solution with H_2_ gas and measuring the open‐circuit potential of the reference electrode versus a polycrystalline platinum electrode in the same electrolyte. The calibrated reference electrode potential fluctuated between 0.695 and 0.701 V_RHE_.

### Ex Situ Electrochemical Characterization—Electrode Preparation

All catalyst inks were prepared using 10.0 mg of catalyst dispersed in 4.00 mL H_2_O, 1.00 mL isopropanol, and 20 µL Nafion‐117 ionomer solution (5 wt. % in lower aliphatic alcohols and water). The commercial catalysts were chosen based on their electrochemical performance. The Comm. 1‐IrO_x_ was a highly active Ir‐based catalyst, while the Comm. 2‐IrO_2_ catalyst was characterized as a crystalline iridium oxide that was less active but more stable than the Comm.1‐IrO_x_ catalyst. To ensure an even dispersion of catalyst particles, the inks were sonicated for 30 min and stirred for a further 30 min. To deposit the catalyst layer onto the micro‐polished glassy carbon electrode disc (5 mm diameter, 0.196 cm_geo_^2^ geometric area), 10 µL of catalyst ink was drop‐cast onto the electrode disc, while stirring the ink, to achieve a final catalyst loading of 100 µg_cat_ cm_geo_^−2^, corresponding to a nominal net iridium loading of 72 µg_Ir_ cm_geo_^−2^ on the electrode. All electrodes were dried in the air.

### Ex Situ Electrochemical Characterization—Electrochemical Measurement Protocol

The catalysts were evaluated for their electrochemical OER activity and stability using a Biologic SP‐300 potentiostat. As a first step, cyclic voltammetry (CV) was used to activate the catalysts and remove contaminants and synthesis residuals from the catalyst surface.^[^ ^35a^ ^]^ For this purpose, the catalysts were cycled between 1.0–1.4 V versus RHE for 10 cycles at a scan rate of 50 mV s^−1^, followed by a further 10 cycles within the same potential range at a scan rate of 10 mV s^−1^. Catalyst activation was then continued using chronoamperometry. The potential was increased stepwise from 1.40–1.48 V versus RHE in 20 mV intervals and held at each step for one minute.^[^ ^18^ ^]^

Chronoamperometry was used to evaluate the OER activity of the catalysts by application of a one‐minute potential hold at 1.500, 1.525, 1.540, and 1.560 V versus RHE. To ensure that (quasi‐)steady‐state conditions were achieved and the measured current was representative of the OER kinetics, the current data of the last 30 seconds of each potential hold were averaged for analysis.^[^ ^2^ ^]^ In addition, cyclic voltammetry was used to evaluate the activity. Cyclic voltammograms were recorded from 1.0–1.6 V versus RHE with a scan rate of 10 mV sec^−1^. For data analysis, the anodic and cathodic scans were averaged to avoid over‐ or under‐estimating the change in current density with potential.

Subsequently, to evaluate the stability, the catalysts were subjected to an accelerated stress test (AST) consisting of chronoamperometry at 1.6 V versus RHE for 4 h. During the chronoamperometric experiment, 85% of the Ohmic drop was automatically/online *iR*‐corrected for by the potentiostat. This chronoamperometric OER activity evaluation was repeated after the AST to assess the activity loss compared to the initial test.

Electrochemical impedance spectroscopy (EIS) was used to determine the solution resistance for Ohmic‐drop correction, which was measured at 1.0 V versus RHE within a frequency range from 200 kHz to 100 mHz. The obtained values of the solution resistance ranged from 5–7 Ω, determined as the high‐frequency intercept of the Nyquist plot with the real axis.

### In Situ Electrochemical Characterization*—*Preparation of Catalyst‐Coated Membranes (CCMs)

All electrodes were fabricated on a Nafion 115 proton exchange membrane (IonPower, USA) to have a 4 cm^2^ active area. For the anodes, catalyst inks were prepared using iridium oxide catalyst powders, isopropyl alcohol (99.9%, Kimix), deionized water (18 MΩ, Millipore), and Nafion D2021 ionomer dispersion (IonPower, USA). The ionomer content in the anodic inks was fixed at 12 wt.%. The ink suspensions were homogenized using a combination of magnetic stirring and ultrasonication. These inks were coated onto cathodes consisting of 0.50 mg_Pt_ cm^−2^ (HyPlat (Pty) Ltd.) using a SonoTek Exacta Coat ultrasonic spray coater (SonoTek Corporation, USA). All anodes were prepared to have an iridium loading of 2.00 mg_Ir_ cm^−2^. The CCM containing the Comm. 2‐IrO_2_, catalyst was procured from HyPlat (Pty) Ltd. and has the same Ir and Pt electrode loadings as our homemade CCM.

### In Situ Electrochemical Characterization—Cell Assembly and Performance Evaluation

The CCMs prepared above were assembled in the wet state between titanium porous transport layers (CURRENTO 2GDL20N‐1.0 (440 × 550 mm), with Pt coated on both sides (thickness of 0.2 µm, Bekaert). The assembly was sealed using an ice‐cube gasket (60 FC‐FKM 200 0.8, Freudenberg), and compressed to 4.0 kN in a 4 cm^2^ test fixture (Fraunhofer ISE). The assembled cells were then tested in single‐cell electrolyzer mode (E20 electrolyzer test station, Greenlight Innovation, Canada). Prior to testing, the assembly was heated for 2 h by flowing water through the cell at 60 °C at a flow rate of 0.1 L min^−1^. Following this, the cell was conditioned for 38 h at a fixed voltage of 2.0 V to activate the catalyst layers adequately. Polarization curves were measured from 0.01 to 3 A cm^−2^ at 60 °C to reduce the possibility of mass transport voltage losses prevalent at higher cell testing temperatures. The cell's high‐frequency resistance (HFR) was measured from 0.01 to 3 A cm^−2^ using a Gamry Reference 3000 and a Reference 30K booster to allow for the conversion of the measured current‐voltage characteristics to iR‐free polarization curves.

## Conflict of Interest

The authors declare no conflict of interest.

## Supporting information



Supporting Information

## Data Availability

The data that support the findings of this study are available from the corresponding author upon reasonable request.
