# Supplementary material for: Perchlorate Fusion–Hydrothermal Synthesis of Nano‐Crystalline IrO2: Leveraging Stability and Oxygen Evolution Activity
Source: Small. 2025 Mar 30;21(20):2412237. doi: 10.1002/smll.202412237 (PMC12087815; doi:10.1002/smll.202412237)
Supplement: Supplementary file 1 — Supporting Information [file SMLL-21-2412237-s001.docx]

Supporting Information

**Perchlorate Fusion–Hydrothermal Synthesis of Nano-Crystalline IrO_2_: Leveraging Stability and Oxygen Evolution Activity**

*Genevieve C. Moss, Tobias Binninger, Ziba S. H. S. Rajan, Bamato J. Itota, Patricia J. Kooyman, Darija Susac, and Rhiyaad Mohamed**

**Section 1.** Estimation of the maximum possible contribution of chloride oxidation to the total electrochemical charge recorded during OER activity testing.

$$m_{IrO_{2}-PFHT catalyst on electrode}=1.99\times{10}^{-5} g_{cat}$$

$$m_{Cl on electrode}=5 wt. \%= 9.96 \times{10}^{-7}g_{Cl}$$

$$n_{Cl}= \frac{m}{M}=\frac{9.96 \times{10}^{-7}g}{35.45 g{mol}^{-1}}=2.81\times{10}^{-8} {mol}_{Cl}$$

$$Charge of 1 mol electrons:96500 C {mol}^{-1}$$

$$Charge of {Cl}^{-}ions=96500 C{mol}^{-1} 2.81\times{10}^{-8} {mol}_{Cl}=2.71 mC$$

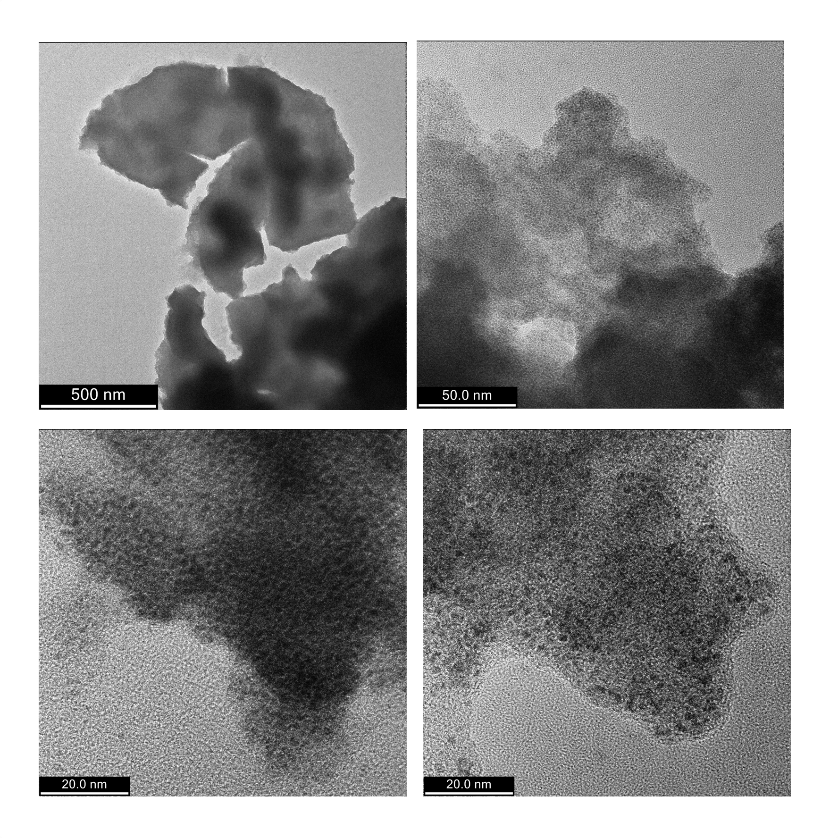


**Figure S1.** TEM images of the synthesis intermediate obtained after the initial calcination step of the H_2_IrCl_6_ precursor with the perchlorate oxidant at 300 °C for 2h. The resulting intermediate was resuspended in H_2_O to obtain a mixture of dissolved iridium salts as well as a black solid component. The solid component imaged here reveals seed IrO_x_ particles which are grown in the subsequent hydrothermal treatment to yield the final IrO_2_–PFHT catalyst.


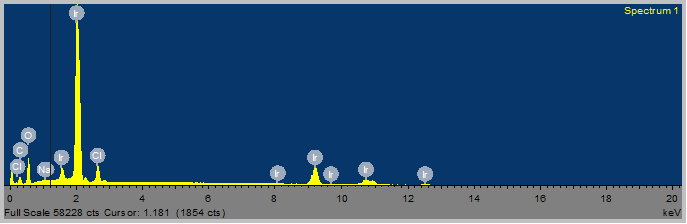


**Figure S2.** EDX spectrum of the final IrO_2_-PFHT catalyst. 5 wt. % Chlorine was detected.


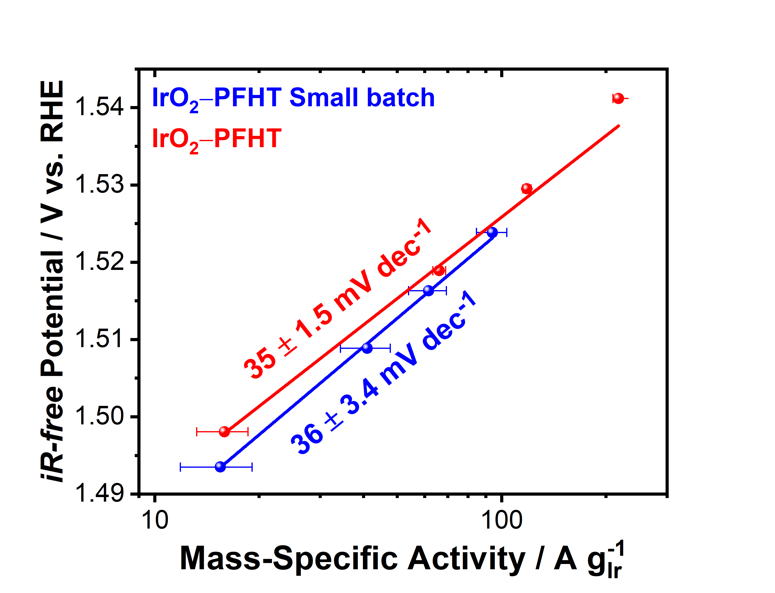


**Figure S3.** Tafel plots of the OER kinetic currents of the small batch IrO_2_-PFHT catalyst against the IrO_2_-PFHT upscaled catalyst. Note that the measurement for the IrO_2_-small batch catalyst was performed in 0.1 м HClO_4_ whilst the upscaled IrO_2_-PFHT catalyst was tested in 0.5 м H_2_SO_4_. Despite the different electrolytes used, the obtained OER activities of both materials were in good agreement, demonstrating the reproducibility of the upscaled synthesis.


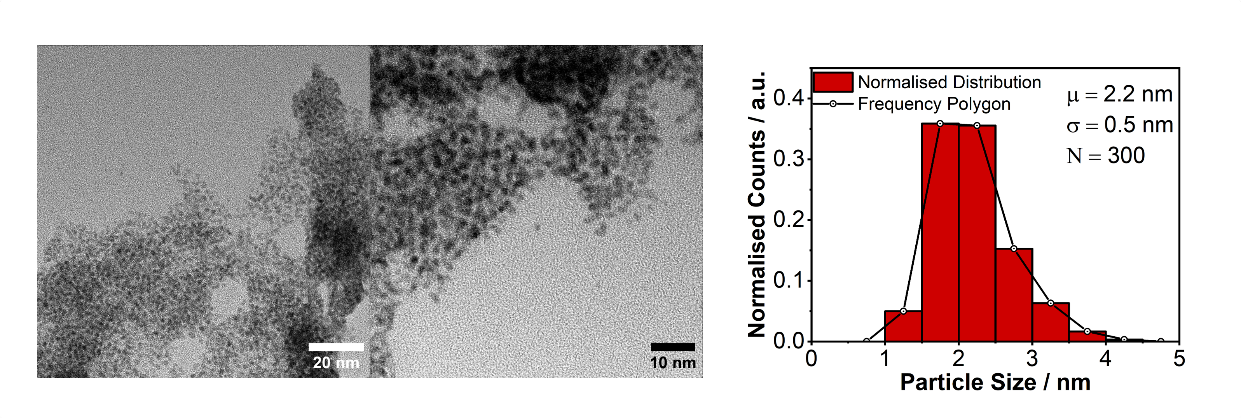


**Figure S4.** TEM images and particle size distribution of the upscaled IrO_2_-PFHT catalyst.


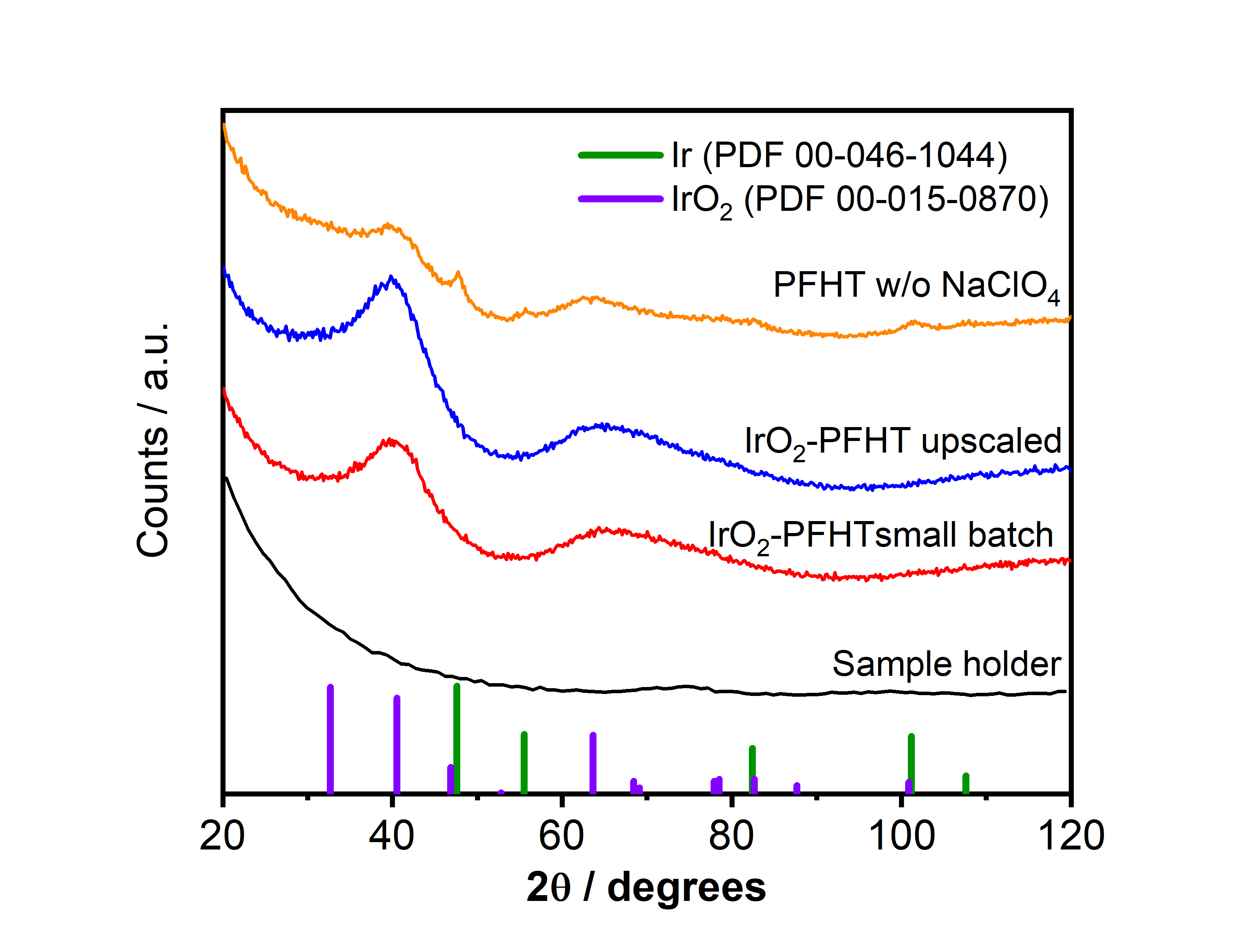


**Figure S5**. X-ray diffractograms of both the upscaled and smaller batch IrO_2_-PFHT catalysts, as well as a sample prepared according to the PFHT method without the use of NaClO_4_ (labeled PFHT w/o NaClO_4_). The pattern of the sample holder is also shown.


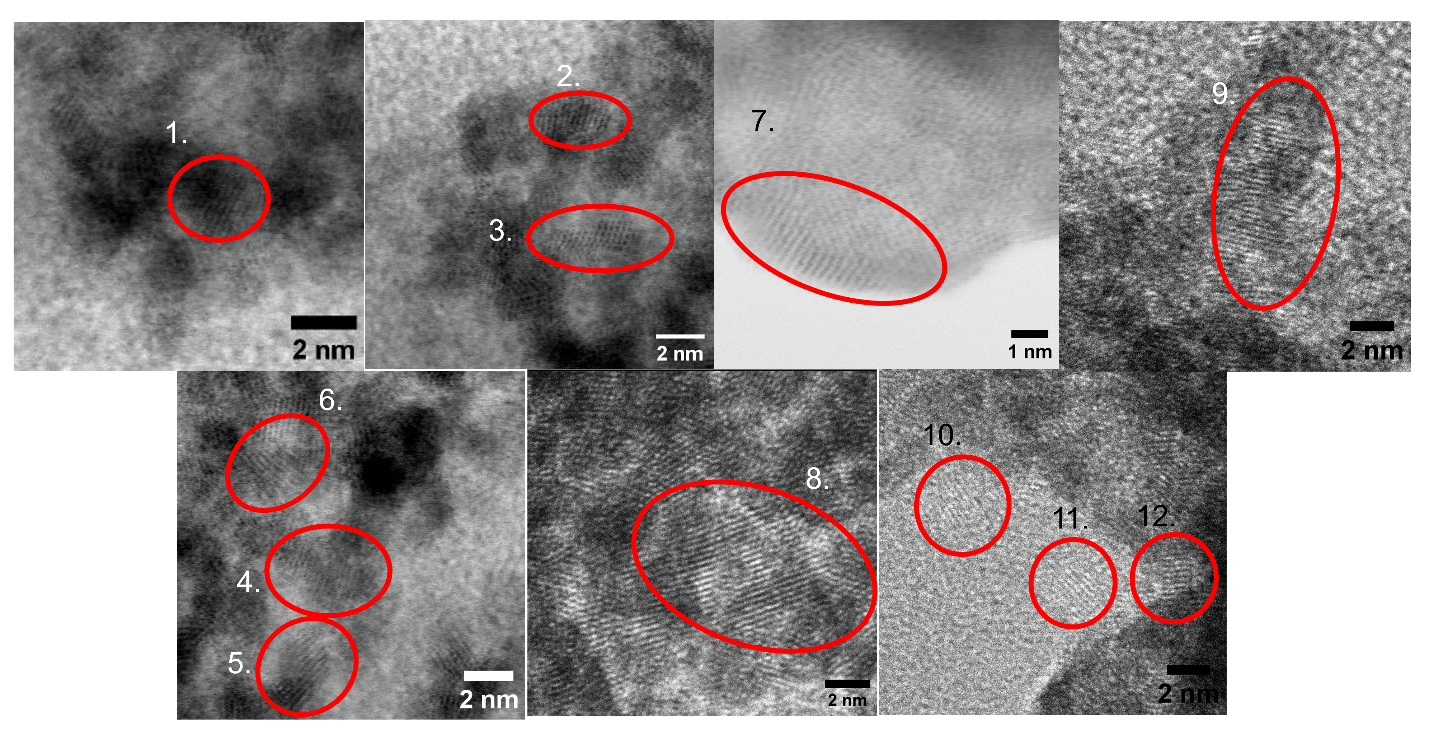


**Figure S6.** TEM images of the IrO_2_-PFHT catalyst particles used for d-spacing measurements.


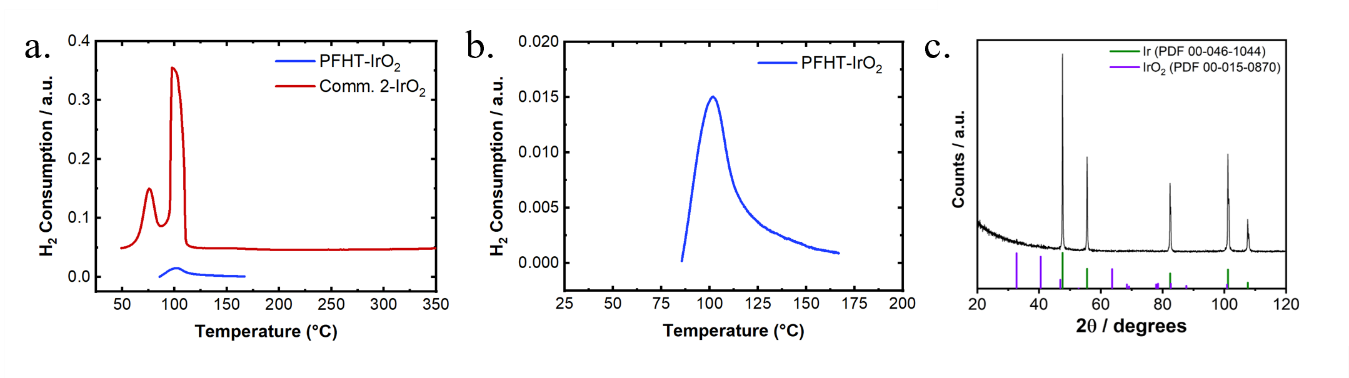


**Figure S7.** (a-b) TPR results of the PFHT-IrO_2_ and Comm. 2-IrO_2_ catalysts. (c) XRD of the IrO_2_-PFHT catalyst post TPR analysis.


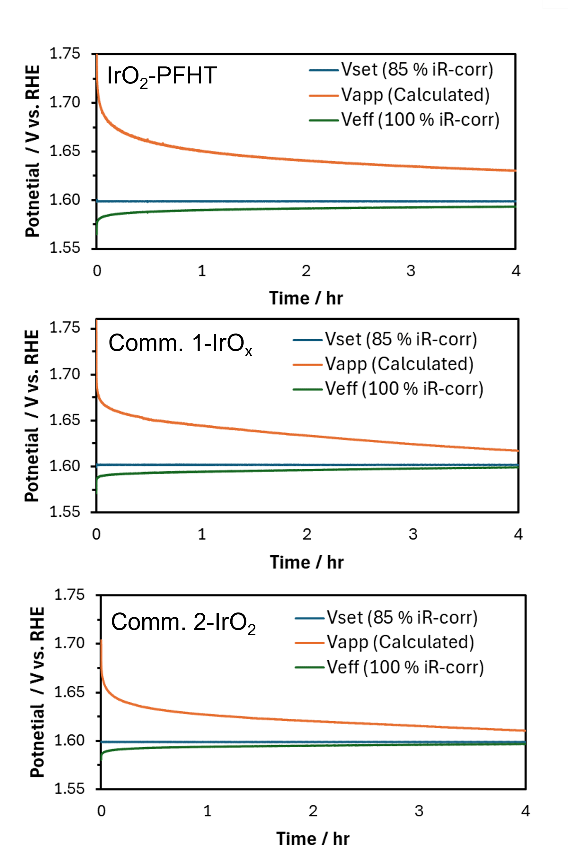


**Figure S8.** Potential vs. time plots of the chronoamperometry for the IrO_2_–PFHT catalyst, the commercial IrO_x_ benchmark (Comm. 1-IrO_x_) and the commercial IrO_2_ (comm. 2-IrO_2_) catalyst where the set potential of 1.6 V vs. RHE (Vset) is online corrected for 85 % of the ohmic iR-drop, shown against the (calculated) actual applied potential (Vapp) and the effective potential at the working electrode after 100 % iR-correction.


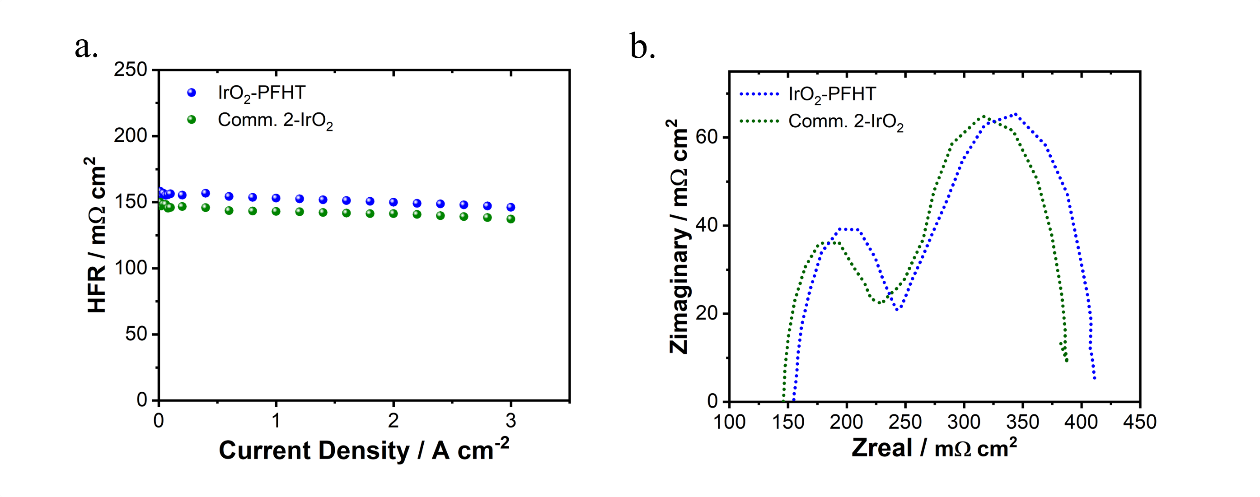


**Figure S9**. (a) High-frequency resistance (HFR) versus the current density of the CCMs. (b) Nyquist plots at 0.1 A cm^–2^.


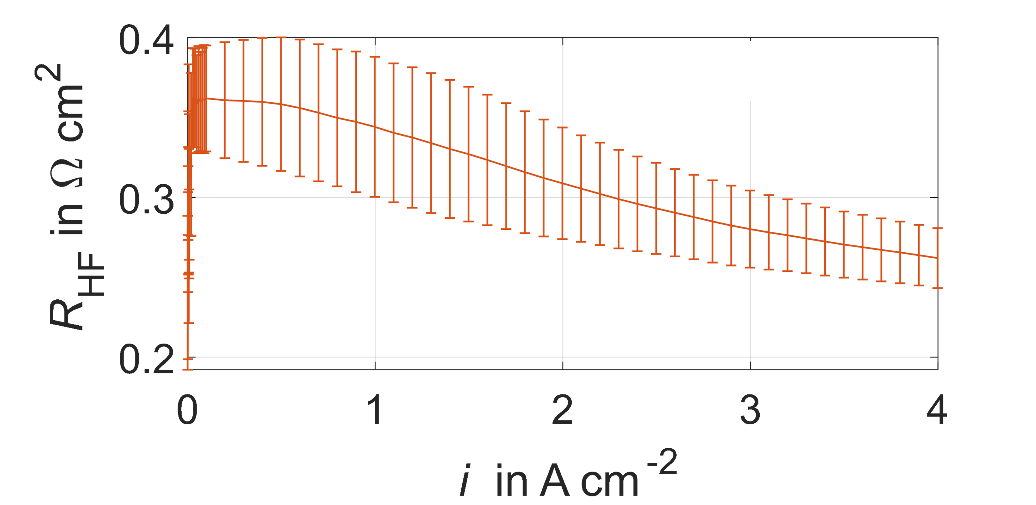


**Figure S10.** High-frequency resistance (HFR) versus current density of a CCM consisting of the Comm. 1-IrO_x_ catalyst at the anode. The high contact resistance in this electrode limited the acquisition of polarization curves to demonstrate *in-situ* performance despite several electrode fabrication attempts at various ionomer loadings.
